# A Nine-Year-Old Girl With Cornelia de Lange Syndrome: A Case Report and Review of the Literature

**DOI:** 10.7759/cureus.74007

**Published:** 2024-11-19

**Authors:** Hanae Bahari, Hind Zahiri, Aziza Elouali, Maria Rkain, Abdeladim Babakhouya

**Affiliations:** 1 Department of Pediatrics, Mohammed VI University Hospital, Faculty of Medicine and Pharmacy, Mohammed I University of Oujda, Oujda, MAR

**Keywords:** child, cornelia de lange syndrome, facial dysmorphia, hirsutism, pediatric case, synophrys

## Abstract

Cornelia de Lange syndrome is a genetic disorder that affects multiple systems. It is characterized by growth delays and psychomotor retardation associated with various anomalies, including hirsutism, facial dysmorphism, cardiac abnormalities, upper-extremity malformations, and gastrointestinal disorders. Early detection and appropriate management of associated disorders are essential for achieving favorable outcomes. We present our first case of Cornelia de Lange syndrome, diagnosed at the age of nine years in the Pediatrics Department of Mohammed VI University Hospital in Oujda, Morocco.

## Introduction

Cornelia de Lange syndrome (CdLS) is a genetically inherited multisystem disorder first described in two infants in 1933 [1]. Although most cases are thought to occur sporadically, familial patterns of inheritance and parental consanguinity are also notable contributing factors [2]. This syndrome is characterized by a range of physical, cognitive, and behavioral features, including a distinctive facial appearance, growth delays, cognitive impairments, behavioral difficulties, and limb abnormalities [3]. The prevalence of CdLS is estimated to be between 1 in 10,000 and 1 in 40,000 live births [4]. While the exact cause remains unclear, five causative genes for CdLS have been identified. Pathogenic variants in the* NIPBL* gene are present in 50-60% of patients, with truncating mutations (nonsense mutations, splice site alterations, and frameshift mutations) linked to more severe phenotypic outcomes. Variants in the *SMC1A*, *SMC3*, *RAD21*, and *HDAC8* genes account for 5-10% of cases and are associated with milder phenotypes, similar to the effects of missense mutations in NIPBL [1]. In its classical form, the condition is easily identifiable based on clinical features; however, genetic testing is frequently employed to affirm the diagnosis [5]. Characteristic facial features include synophrys, arched eyebrows, long eyelashes, a short-upturned nose, thin downturned lips, and micrognathia [6].

The purpose of this article is to provide a case report of a nine-year-old girl diagnosed with CdLS, emphasizing the importance of early diagnosis, a multidisciplinary approach, and consistent monitoring for affected patients.

## Case presentation

**Figure 1:**
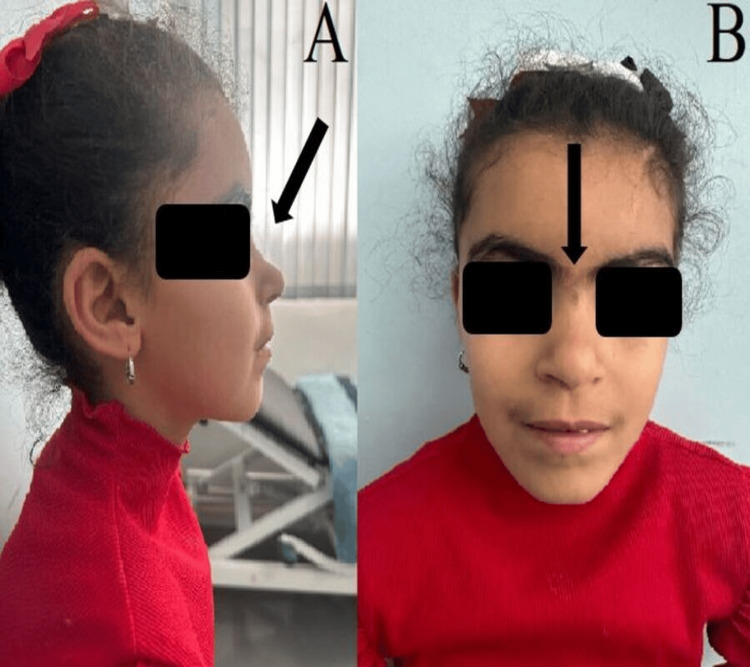
A nine-year-old girl with facial features of Cornelia de Lange syndrome.

We present the case of a nine-year-old girl who was referred to the Pediatric Department due to growth retardation and psychomotor delays. She was delivered at term via spontaneous vaginal delivery to non-consanguineous parents, from a (mother gravida 3, para 3, 30 years old), without prenatal care or ultrasounds, and with no significant pathological history. Clinical examination revealed a malformative syndrome characterized by well-defined arched eyebrows, synophrys, long eyelashes, a short nose, a concave nasal ridge, anteverted nares, hirsutism, and a thin upper lip (Figure 1). The patient exhibited global developmental delays, particularly in speech, which evolved to include intellectual delays and autistic traits, alongside severe short stature (115 cm, −2.00 standard deviations) and underweight (18 kg, −2.00 standard deviations). No limb malformations were observed. Hearing loss was noted without any additional malformations. Written informed consent to include these images in the published article was obtained from the patient's legal guardian.

The laboratory tests, including complete blood count, biochemical parameters, urinalysis, and growth hormone dosage, all returned normal results.

**Figure 2:**
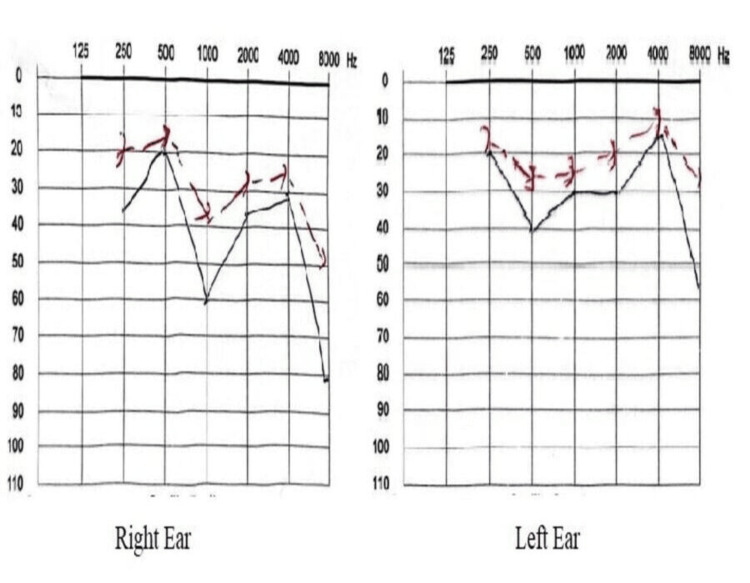
Audiometry results showing congenital mixed hearing loss in a nine-year-old girl with Cornelia de Lange syndrome. The red line indicates bone conduction, and the black line indicates air conduction.

An audiometry test was conducted, which revealed a congenital mixed hearing loss (Figure 2).

The ophthalmological evaluation revealed severe binocular functional amblyopia, secondary to a refractive error, with visual acuity measured using the Snellen chart at 5 meters: right eye 6/10 and left eye 4/10. There were no abnormalities during a neurological evaluation using cerebral magnetic resonance imaging (MRI). An echocardiogram and a renal ultrasound were performed, both of which returned normal results.

Following these findings, the diagnosis of CdLS was established according to the criteria defined by the CdLS Foundation [7], without reliance on molecular biology, with a clinical score of 10 (of which 4 criteria were cardinal) evoking a CdLS. Chromosomal analysis performed on peripheral blood using conventional techniques revealed a normal female karyotype (46, XX).

Multidisciplinary care, focused on the specific anomalies presented by the patient, was provided with regular follow-up. This involved a team of specialists, including a pediatrician, geneticist, ophthalmologist, otorhinolaryngologist, speech-language pathologist, and social worker, each responding to the patient's individual needs. The collaborative approach ensured comprehensive management, encompassing strategies for hearing and vision correction, with appropriate interventions implemented and continuous monitoring to assess outcomes.

## Discussion

CdLS is a complex congenital disorder characterized by intellectual disability, and its etiology remains unclear. There is no documented predilection regarding age, race, or sex. Estimating the overall prevalence of CdLS is challenging due to the unidentified proportion of milder cases. Nevertheless, it is estimated to occur in approximately 1 in 10,000 to 1 in 40,000 live births, with around one-third of affected individuals being born prematurely [4,8].

The diagnosis of CdLS is established when a patient displays characteristic clinical features and/or when a pathogenic variant is identified through molecular genetic testing [8,9]. In our case, the diagnosis was based on clinical presentation alone, as molecular genetic testing was not available at our hospital. The patient’s clinical score of 10, which included 4 cardinal criteria, was suggestive of CdLS. However, KBG syndrome, a genetic condition caused by mutations in the *ANKRD11 *gene, was also considered in the differential diagnosis due to its similarities with CdLS [9]. Both conditions share features such as intellectual disability, short stature, and distinct facial dysmorphology, as well as hearing loss, which was present in our patient. Despite these similarities, KBG syndrome is typically associated with macrodontia, a feature absent in our patient.

The diagnostic criteria for CdLS are not well-defined. Key features associated with the syndrome include distinctive facial abnormalities such as synophrys, elongated eyelashes, microcephaly, and anteverted nostrils. Cardiovascular anomalies can include various conditions such as ventricular or atrial septal defects, aortic or pulmonary stenosis, tetralogy of Fallot, atrioventricular canal defects, single ventricle, aorto-pulmonary window, and truncus arteriosus communis. Furthermore, upper limb anomalies may manifest as ectrodactyly and monodactylism. Gastrointestinal complications can present as diaphragmatic hernia, and other notable abnormalities include musculoskeletal malformations and intrauterine growth retardation [10]. Hearing loss is highly prevalent among children with CdLS, affecting more than 80% of cases, as observed in our case report. However, the presence of intellectual disability and the early onset of symptoms can complicate the assessment of hearing. Therefore, it is crucial to employ objective testing methods to accurately evaluate hearing capabilities in these patients [11,12]. Renal abnormalities, particularly vesicoureteral reflux, occur in approximately 12% of cases, underscoring the importance of early renal and cardiological screening in suspected cases of CdLS to facilitate timely diagnosis and intervention [9,13,14]. Musculoskeletal and genital abnormalities are also commonly seen in this syndrome. These include hip abnormalities such as dislocation or dysplasia (10%), scoliosis, tight Achilles tendons, and the development of bunions. Genital anomalies can include hypoplastic external male genitalia (57%), small labia majora, undescended testes (73%), and hypospadias (33%). Ophthalmologic manifestations are also prevalent, with approximately 50% of cases showing eye-related issues. These include myopia (58%), ptosis (44%), blepharitis (25%), epiphora (22%), microcornea (21%), strabismus (16%), and nystagmus (14%) [15,16]. Seizures, particularly partial epilepsy, are common in CdLS, often starting before age 2 and responding well to standard treatment. Other neurological symptoms include autonomic nervous system abnormalities, sensory deficits, brain structural changes, and, in rare cases, dystonia, catatonia, and spinal cord abnormalities [7].

A study by Marchisio et al. found that 26 (59%) out of 44 pediatric patients with CdLS had conductive hearing loss [15,16]. Kim et al. conducted the first evaluation of ophthalmologic issues in a case of CDLS. They described an 18-year-old female with CDLS presenting with superficial keratitis, entropion, ptosis, high myopia, lacrimal cutaneous fistula, and distinctive facial features [17]. Additionally, Grau Carbó et al. have reported cases with clinical features characteristic of CDLS [18].

Molecular diagnostic criteria have been established to identify abnormalities in genes associated with chromatin regulation, particularly those involved in cohesion complexes. CdLS is mostly caused by mutations in the NIPBL, SMC1L1, and SMC3 genes. Research focusing on genotype-phenotype correlations indicates notable differences in the degree of growth and developmental delays between patients with mutations and those without [14,19]. While molecular genetic testing is recommended for a definitive diagnosis, it may not always be readily accessible or affordable in all healthcare settings, including our own.

When a diagnosis of CdLS is confirmed, a multidisciplinary approach is adopted. The medical and surgical management of CdLS focuses on preventing treatable complications and enhancing developmental potential. The emphasis is on managing significant malformations and their treatment, alongside continuous monitoring. Standard evaluations, such as echocardiography and renal ultrasound, are advised for all children with CdLS. Additionally, imaging of the central nervous system should be conducted if neurological symptoms are present. Timely intervention is crucial for addressing congenital heart conditions, urinary tract issues, feeding difficulties, developmental delays, and sensory impairments, including hearing and vision deficits. Regular follow-up, ideally on an annual basis, is recommended for all infants and children diagnosed with CdLS [13].

Life expectancy in individuals with CdLS is estimated to be 10-20 years shorter than that of the general population, with variations depending on the severity of the condition and the presence of complications. The leading causes of death are respiratory and gastrointestinal diseases, untreated cardiac defects, and seizures [20].

## Conclusions

CdLS is often strongly suspected based on clinical signs, particularly when key features such as facial dysmorphisms, growth delays, and intellectual disability are present. Consequently, recognizing these clinical characteristics is crucial for early diagnosis and timely intervention, which are essential to support normal growth and development in affected children. Despite significant strides in understanding CdLS, further research is necessary to clarify the genotype-phenotype correlations and to develop more targeted therapeutic interventions.
